# Treatment-related mortality with aflibercept in cancer patients

**DOI:** 10.1007/s00228-014-1715-9

**Published:** 2014-07-25

**Authors:** Josep Tabernero, Simon Hitier, Eric Derobert, Eric Van Cutsem

**Affiliations:** 1 Vall d’Hebron University Hospital and Vall d’Hebron Institute of Oncology (VHIO), Barcelona, Spain; 2 Sanofi Biostatistics, Oncology R&D, Vitry-sur-Seine, France; 3 University Hospitals Leuven and KU Leuven, Leuven, Belgium

We read with interest ‘Treatment-related mortality with aflibercept in cancer patients” by Wei-Xiang Qi and colleagues in the April 2014 issue of your journal [1], in which they conclude that the use of aflibercept is associated with an increased risk of fatal adverse events (FAEs) compared with controls. It is our opinion that this meta-analysis has some important methodological flaws.

The analysis was conducted using the published data for 3,060 patients, with a range of solid tumors. In 954 patients who received single-agent aflibercept and had data available for analysis, the authors estimated the overall incidence of FAEs to be 5.1 % (95 % CI 3.8–6.8) [1]. However, the authors erroneously included the VENICE trial [2] in their estimate of the incidence of FAEs with single-agent aflibercept. The phase III VENICE trial compared docetaxel and prednisone plus either aflibercept or placebo for the treatment of metastatic castration-resistant prostate cancer and therefore is not informative for single-agent aflibercept. Also, in the VENICE trial, the aflibercept dose was 6 mg/kg and not the 4 mg/kg dose approved for use in patients with metastatic colorectal cancer (mCRC) who have progressed on prior oxaliplatin-containing therapy. Another important point is that the methodology used for calculating the incidence of FAEs may be inappropriate. By using the logit transformation (extremely unusual when estimating a single proportion), the authors implicitly assigned weights that were not proportional to the number of patients. Consequently, studies with high FAE rates are over-weighted. The exclusion of the VENICE trial and the subsequent assignment of appropriate weights result in an estimate of the incidence of FAEs of 3.2 % (95% CI 1.6–5.6) for single-agent aflibercept.

Also, in an attempt to determine the specific contribution of aflibercept to the development of FAEs, the authors calculated the odds ratio (OR) for FAEs from four randomized clinical trials comparing aflibercept with controls in 2,715 patients. Most surprisingly, they excluded from their analysis the pivotal, randomized, VELOUR trial, in patients with mCRC who were resistant to or had progressed on a prior oxaliplatin-containing regimen [3], which led to the only approved indication for aflibercept. Furthermore, the authors extracted incorrect FAE values for the phase III VITAL study [4]. The correct values for FAEs in the VITAL study [4] are 32 FAEs in the aflibercept group versus 18 in the control group, and not 11 versus 1. For all FAE recalculations that included the VITAL study, we used the data from the referenced publication.

Inclusion of the VELOUR study in the estimates of FAEs results in an OR of 1.516 (95 % CI 1.124–2.053, one-sided *p* = 0.0028). Taking into account only the studies using the approved 4 mg/kg dose and including the pivotal VELOUR study [3], the OR is 1.289 (95 % CI 0.836–2.001, one-sided *p* = 0.1354), corresponding to an increased risk of FAEs of approximately 29 %. For the two randomized controlled trials using the higher 6 mg/kg dose of aflibercept (2,137 patients) [2, 4], the OR is 1.767 (95 % CI 1.156–2.734, one-sided *p* = 0.0037), corresponding to an increase in the relative risk of FAEs of 77 %.

We would like to reiterate that in this meta-analysis, the studies used to estimate the incidence of FAEs with aflibercept in cancer patients are all outside the labeled indication for aflibercept. Also, the exclusion of the VELOUR trial renders the results of this meta-analysis incomplete and prone to misinterpretation, although the authors conclude that the use of aflibercept remains justified in its approved indication in patients with mCRC. Importantly, and as reported previously for VELOUR [3] and other clinical studies [5], the safety profile associated with combining aflibercept with chemotherapy is similar to that seen with other anti-VEGF agents, with no new toxicity signals observed.
